# Simplified ultrasound protocol for the exclusion of clinically significant carotid artery stenosis

**DOI:** 10.1080/03009734.2016.1201177

**Published:** 2016-06-29

**Authors:** Dominika Högberg, Demosthenes Dellagrammaticas, Björn Kragsterman, Martin Björck, Anders Wanhainen

**Affiliations:** a Department of Surgical Sciences, Section of Vascular Surgery, Uppsala University, Uppsala, Sweden;; b Department of Surgery, NU Hospital Organization, Trollhättan, Sweden

**Keywords:** Carotid stenosis, screening, ultrasound

## Abstract

**Objectives:**

To evaluate a simplified ultrasound protocol for the exclusion of clinically significant carotid artery stenosis for screening purposes.

**Material and methods:**

A total of 9,493 carotid arteries in 4,748 persons underwent carotid ultrasound examination. Most subjects were 65-year-old men attending screening for abdominal aortic aneurysm. The presence of a stenosis on B-mode and/or a mosaic pattern in post-stenotic areas on colour Doppler and maximum peak systolic velocity (PSV) in the internal carotid artery (ICA) were recorded. A carotid stenosis was defined as The North American Symptomatic Carotid Endarterectomy Trial (NASCET) >20% and a significant stenosis as NASCET >50%. The kappa (κ) statistic was used to assess agreement between methods. Sensitivity, specificity, positive predictive (PPV), and negative predictive (NPV) values were calculated for the greyscale/mosaic method compared to conventional assessment by means of PSV measurement.

**Results:**

An ICA stenosis was found in 121 (1.3%) arteries; 82 (0.9%) were graded 20%–49%, 16 (0.2%) were 50%–69%, and 23 (0.2%) were 70%–99%. Eighteen (0.2%) arteries were occluded. Overall, the greyscale/mosaic protocol showed a moderate agreement with ICA PSV measurements for the detection of carotid artery stenosis, κ = 0.455. The sensitivity, specificity, PPV, and NPV for detection of >20% ICA stenosis were 91% (95% CI 0.84–0.95), 97% (0.97–0.98), 31% (0.26–0.36), and 97% (0.97–0.97), respectively. The corresponding figures for >50% stenosis were 90% (0.83–0.95), 97% (0.97–0.98), 11% (0.08–0.15), and 100% (0.99–1.00).

**Conclusion:**

Compared with PSV measurements, the simplified greyscale/mosaic protocol had a high negative predictive value for detection of >50% carotid stenosis, suggesting that it may be suitable as a screening method to exclude significant disease.

## Introduction

Carotid atherosclerotic disease accounts for 20% of ischemic stroke and is the most common cause of stroke in middle-aged patients (1). Several publications indicate that appropriate medical therapy and risk factor control prevent the development of cerebrovascular symptoms due to carotid stenosis (2–5). Hence, screening for carotid stenosis has been discussed during the last decade; although current vascular guidelines do not recommend unselected population screening (6–8), there are recommendations for screening high-risk adults with multiple cardiovascular risk factors (9,10). In a recent study of the Swedish population we observed that only approximately 40% of individuals with screening-detected asymptomatic carotid stenosis were on preventive medication with statins and/or antiplatelet agents (11).

**Figure 1.**
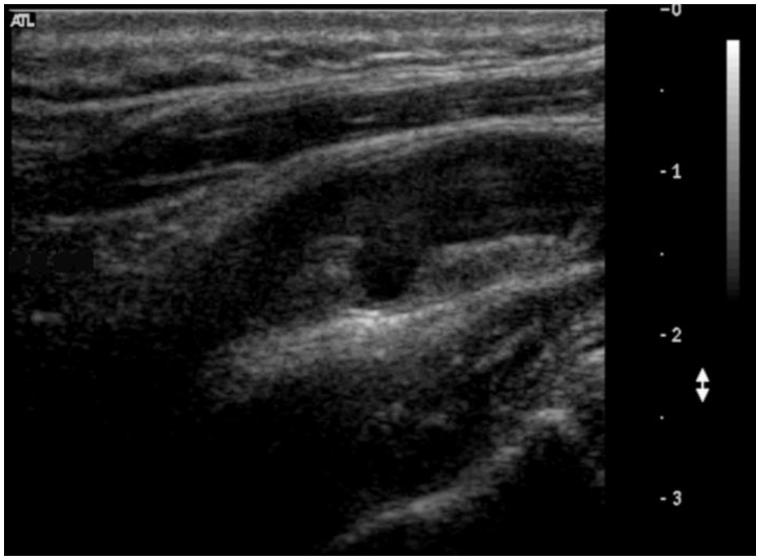
Carotid artery with stenosis on greyscale.

**Figure 2.**
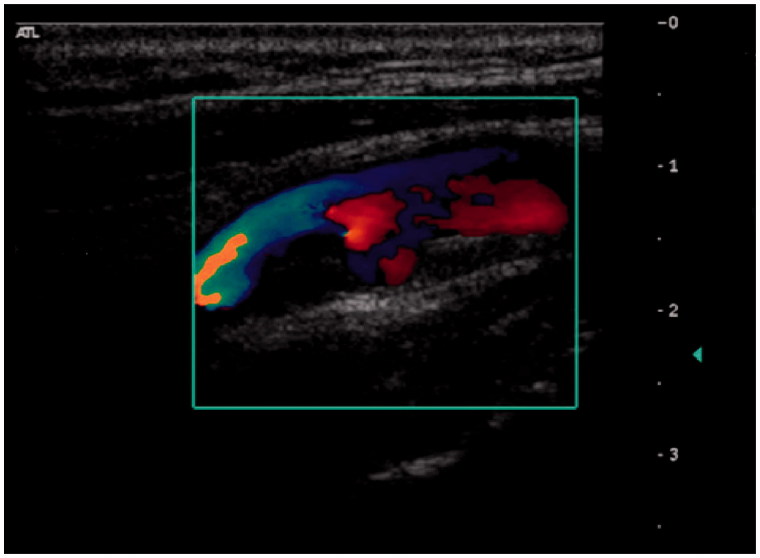
Carotid artery with stenosis and mosaic pattern on colour Doppler.

Duplex ultrasonography (DUS) is the standard diagnostic method for carotid artery stenosis and includes three modalities: B-mode (greyscale), colour Doppler evaluation, and velocity measurements. B-mode allows measurements of intima-media thickness (IMT) and characterization of atherosclerotic plaque morphology (Figure 1). Colour Doppler allows for visualization of flow abnormalities such as turbulence related to the presence of stenosis, which gives rise to a characteristic ‘mosaic’ pattern (Figure 2). However, it is spectral analysis of the Doppler waveform together with measurement of blood flow velocity which is the main parameter used for grading the severity of carotid stenosis (12,13). Velocity measurements have certain technical aspects that are important for accurate assessment, such as correct positioning of the sample volume, complete sampling through an area of stenosis, and obtaining a correct Doppler angle of insonation. A full carotid duplex ultrasound protocol is therefore highly operator-dependent and can be time-consuming.

The aim of this study was to examine whether a simplified DUS protocol consisting of assessment for presence of stenosis on B-mode and/or mosaic patterns on colour Doppler (the greyscale/mosaic method) could be used as a rapid and reliable screening method for the exclusion of clinically significant carotid stenosis.

## Material and methods

A total of 4,748 subjects were included in the study; 4,646 were healthy 65-year-old men attending a population-based carotid screening programme during 2007–2009, taking the opportunity of the abdominal aortic aneurysm screening programme that invites all 65-year-old men for ultrasound screening in Uppsala since 2006, with an attendance rate of 86% (14,15). The results of this carotid screening study have been published previously (11). We also included 102 consecutive patients undergoing carotid DUS in 2012 for symptomatic cerebrovascular disease to increase the number of patients with carotid artery stenosis, since a greater number of ‘events’ facilitates comparisons between the evaluated methods.

The DUS examinations were carried out at the vascular laboratory at Uppsala University Hospital by three experienced (>3 years of experience in vascular ultrasound) ultrasound technicians. Examinations were conducted either with an Acuson Sequoia system (Acuson, Mountain View, CA, USA), using an L9-4 MHz linear transducer or a Philips iU22 system (Philips Ultrasound, Bothell, WA, USA), using an L9-3 MHz linear transducer. A maximum insonation angle of 60° to the vessel was applied in all examinations. The carotid arteries on both sides were evaluated in both transverse and longitudinal projection for the presence of a visible significant narrowing on B-mode greyscale image and the presence of mosaic pattern on colour Doppler image. Internal carotid artery (ICA) peak systolic velocity (PSV) was then measured in the narrowest segment of the vessel as indicated by B-mode duplex ultrasound and/or colour flow changes. The degree of stenosis was determined according to the NASCET method modified by Jogestrand et al. (16–18), which is the standard used for the accreditation of vascular laboratories in Sweden (Table 1) (18). DUS examinations were performed with the standard presets for carotid ultrasound for each machine. The technician made further optimizations in cases of difficult morphology. The vessels were first examined for presence of stenosis signs with greyscale image and colour Doppler, and the findings were registered into a standardized form, after which the ICA PSV measurements were made and registered. Both the greyscale/mosaic method and PSV measurements were performed by the same technician without blinding. An interobserver variability analysis was performed on subjects from the same ongoing screening programme in which 36 arteries were included with both normal findings and atherosclerosis.

**Table 1.** Standard criteria for grading carotid stenosis in Sweden (16–18).

Systolic maximal velocity		Degree of stenosis	
Angle <45°	Angle 55–60°	ECST	NASCET
<1.1 m/s	<1.3 m/s	<50%	<20%
1.1–1.6 m/s	1.3–2.2 m/s	50%–69%	20%–49%
1.7–2.0 m/s	2.3–3.1 m/s	70%–79%	50%–69%
≥2.1 m/s	≥3.2 m/s	80%–99%	70%–99%
No signal	No signal	Occlusion	Occlusion

ECST: The European Carotid Surgery Trial.

Greyscale and/or mosaic pattern findings were compared with presence of stenosis determined by PSV. Statistical analysis was carried out with SPSS (PC version 20.0, SPSS, Chicago, IL, USA) and the Vassar Stats website for statistical computation (vassarstats.net). The κ statistics were used to assess agreement between methods, and 95% confidence intervals (CI) were calculated. The levels of agreement were defined as: κ < 0.20 poor, 0.21 < κ < 0.40 fair, 0.41 < κ < 0.60 moderate, 0.61 < κ < 0.80 good, and κ ≥ 0.80 very good agreement (19). *P* < 0.05 was considered statistically significant. Sensitivity, specificity, positive predictive value (PPV), and negative predictive value (NPV) were calculated with 95% CI. All subjects gave informed consent prior to the investigation. The regional ethical review board in Uppsala approved the study (EPN dnr 2007/053).

## Results

A total of 9,493 carotid arteries in 4,748 subjects were examined. Three subjects underwent a unilateral examination only. The screening group consisted of 4,646 65-year-old men in whom the prevalence of ICA stenosis was 1.9%. The symptomatic group consisted of 56 men and 46 women with a mean age of 67 years (SD ±12) in whom the prevalence of ICA stenosis was 17%.

An ICA stenosis, diagnosed by means of PSV measurement (NASCET definition), was found in 121 (1.3%) arteries; 82 (0.9%) were graded 20%–49% (mild), 16 (0.2%) were 50%–69% (moderate), and 23 (0.2%) were 70%–99% (severe). Eighteen (0.2%) arteries were occluded.

The total time taken to examine both carotid bifurcations using the greyscale/mosaic protocol ranged between two and three minutes. Moderate agreement was observed between the two methods, with κ = 0.455 (95% CI 0.399–0.511), *P* < 0.001. For the detection of >20% ICA stenosis the greyscale/mosaic pattern method had a sensitivity of 91% (95% CI 0.84–0.95) and a specificity of 97% (0.97–0.98). The PPV was 31% (0.26–0.36), and the NPV was 97% (0.97–0.97). For the detection of >50% ICA stenosis the sensitivity was 100% (95% CI 0.89–1.0), the specificity 97% (95% CI 0.96–0.97), the PPV was 11% (95% CI 0.08–0.15), and the NPV was 100% (95% CI 0.99–1.0). The greyscale/mosaic method detected all significant stenoses >50% but missed a few <50% stenoses; however, most of these were borderline with an ICA PSV of <2.0 m/s (Table 2). Interobserver agreement was very good, with κ = 0.8 for both mosaic and greyscale evaluation and κ = 1.0 for PSV.

**Table 2.** Contingency table of ultrasound outcome.

	ICA stenosis defined by PSV measurement (NASCET)					
	Normal	20%–49%	50%–69%	70%–99%	100%	Total
ICA stenosis by greyscale/mosaic measurement						
Yes	238	71	16	23	4	352
No	9116	11	0	0	14	9141
Total	9354	82	16	23	18	9493

## Discussion

Recent data (2–4,20) clearly indicate the benefit of current best medical therapy in the management of patients with moderate and severe asymptomatic carotid artery stenosis in order to reduce stroke risk as well as overall cardiovascular risk. In a natural history study Nikolaides et al. reported that a considerable number of neurological events occur in patients with low-grade stenosis (<60%, NASCET criteria) (21). There is also evidence that best medical treatment (BMT) affects the intima-media thickness progression and stroke risk, which suggests that individuals with mild and moderate stenosis also could benefit from preventive treatment (22). In a recent population study we observed that most men with a screening-detected asymptomatic carotid stenosis had no other clinical manifestation of atherosclerosis. In fact, only about 40% of individuals in the whole group were receiving a statin and an antiplatelet agent at the time of screening (11). As a consequence, carotid artery screening has a potential role in identifying individuals at risk and enabling the institution of best medical therapy and appropriate follow-up for this cohort, including a small number even considered for operative intervention. Several issues need to be resolved, however, before screening for asymptomatic carotid stenosis can be advocated. Many of those are included in the World Health Organization guidelines on principles and practice of screening for disease published in 1968 (23); one is the need for a suitable screening method.

Ultrasound is a non-invasive and readily available diagnostic tool that already serves as an excellent screening method in other clinical contexts (e.g. screening for aortic aneurysms). Several definitions and grading methods of an ICA stenosis exist, all based on velocity measurements such as ICA PSV (12). Velocity measurements have certain technical aspects that are important for accurate assessment, such as correct positioning of the sample volume, complete sampling through an area of stenosis, and obtaining a correct Doppler angle of insonation (≤60°). To handle these technical aspects is investigator-dependent (24). While the preoperative diagnostic analysis should be precise because of its utmost importance for the indication for surgery (25–28), a screening duplex scan does not have to be as precise. Instead, it should preferentially be a method that excludes healthy individuals as quickly as possible and, most importantly, identifies the few with a potentially clinically significant stenosis, who can later be examined with higher precision. In a screening setting, a simplified carotid protocol as a primary evaluation may reduce the cost associated with the examination and therefore be preferred compared to a preoperative diagnostic tool. In this context, a simplified DUS protocol may allow the training of less specialized technicians who might concentrate on screening assessments, whilst more experienced technicians might continue to provide a thorough diagnostic assessment.

The present study showed that carotid artery stenosis could be excluded by means of a simplified DUS protocol, with a high negative predictive value (NPV) of 100%. The overall agreement with ICA PSV measurements was moderate, and the greyscale/mosaic pattern method tended to overestimate the number of stenoses. The accuracy of detection of mild stenosis (20%–49%) was very good, and the identification of moderate (50%–69%) and severe (70%–99%) stenoses was excellent.

To our knowledge there are no recent population-based studies that evaluate a simplified screening method for carotid stenosis using ultrasound. Our study, however, is consistent with the study of Hallam et al. published in 1989, who showed in a double-blind comparison complete agreement between colour-flow assessment only and a full DUS assessment in 91% of cases (29). Interestingly, Hallam et al. also found the same κ value as in the present report evaluating the interobserver variability. Another quick carotid scan method was evaluated by Lavenson among 500 consecutive carotid ultrasound patients in 2004; they found a sensitivity of 93% and a specificity of 87% when comparing with a complete carotid ultrasound (30).

The present study was a prospective population-based study with a predefined protocol in which greyscale/mosaic and PSV were measured simultaneously. One important and unforeseen limitation was the low prevalence of significant stenosis in the general population. A group of symptomatic patients was therefore added to the screening cohort to gain more power to the analysis of the simplified DUS method. Before the use of this method as a routine screening protocol can be proposed, there are a few factors that must be taken into consideration. First, our study was not blinded, and observer bias is highly possible. In addition, all technicians performing the assessments were experienced in carotid ultrasound and thus may have been more likely to recognize the hallmark signs of carotid stenosis during assessment with B-mode and colour Doppler. A natural next step in testing this protocol would be to assess how operators with limited previous experience in DUS of the carotid arteries perform compared to experienced ultrasound technicians. To minimize the risk of missing a significant carotid stenosis (in particular with unexperienced operators or in difficult anatomy) subjects with uncertain findings should be referred for a complete carotid ultrasound. Such a study design would probably be more representative of a screening setting, since many abdominal aortic aneurysm screening programmes use less experienced operators to examine the aorta (14,31,32).

It might be concluded that although the greyscale/mosaic protocol showed only moderate agreement with conventional DUS assessment for the detection of carotid stenosis, we observed a NPV of 100%, suggesting that this method may be appropriate as a screening tool. Further studies are required to determine the generalizability of the technique.
